# Iron homeostasis as a cell detoxification mechanism in *Mesorhizobium qingshengii* J19 under yttrium exposure

**DOI:** 10.3389/fmicb.2024.1467386

**Published:** 2024-10-04

**Authors:** Carina Coimbra, Paula V. Morais, Rita Branco

**Affiliations:** University of Coimbra, Centre for Mechanical Engineering, Materials and Processes, ARISE, Department of Life Sciences, Coimbra, Portugal

**Keywords:** RNAseq, transcriptional response, Y stress, Oxidative Stress, iron homeostasis

## Abstract

Yttrium (Y), an important rare earth element (REE), is increasingly prevalent in the environment due to industrial activities, raising concerns about its toxicity. Understanding the effects of Y on microorganisms is essential for bioremediation and biorecovery processes. This study investigates how *Mesorhizobium qingshengii* J19, a strain with notable resistance to Y, manages iron homeostasis as a detoxifying mechanism under Y stress. Using comparative genomic and transcriptomic analyses, we explored the gene expression profile of strain J19 to identify the mechanisms underlying its high Y resistance and effective Y removal from the medium. Genome-wide transcriptional profiling revealed 127 significantly differentially expressed genes out of 6,343 under Y stress, with 36.2 % up-regulated and 63.8 % down-regulated. Notably, Y exposure significantly affects cellular iron homeostasis and activates arsenic detoxifying mechanisms. A key finding was the 7.6-fold up-regulation of a TonB transporter gene, indicating its crucial role in Y detoxification. Real-time PCR (RT-PCR) analysis of the selected gene confirmed the accuracy of RNA sequencing results. Further validation showed that iron supplementation mitigates Y-induced growth inhibition, leading to reduced ROS production in strain J19. This study elucidates the molecular mechanisms by which strain *M. qingshengii* J19 adapts to Y stress, emphasizing the importance of iron in controlling ROS and protecting against Y toxicity. It also highlights critical pathways and adaptive responses involved in the strain’s resilience to metal stress.

## 1 Introduction

Environmental pollution from toxic metals, particularly in natural water sources, has become a significant concern, primarily due to extensive mining and processing activities (Maynaud et al., 2013; Choudhary and Sar, 2016; Igiri et al., 2018; Maertens et al., 2020; Alotaibi et al., 2021). Among the metals released into the environment, the element yttrium (Y) from the rare earth element (REE) group is of considerable industrial and economic value due to its luminescence, strength, and magnetic properties. Although Y is considered a non-essential for living organisms, its role in organisms and the metabolic mechanisms of Y-cell interactions are poorly studied.

Metals are generally essential for organisms, performing critical roles in cellular metabolism, such as stabilizing macromolecular cell structures, catalyzing biochemical reactions, and acting as enzyme cofactors (Hu et al., 2005; Chandrangsu et al., 2017; Wehrmann et al., 2019). However, at high concentrations, metals can become toxic, causing disruptions in protein structure or function, DNA damage, and increased production of reactive oxygen species (ROS), leading to oxidative stress (Choudhary and Sar, 2016; Wehrmann et al., 2019; Alotaibi et al., 2021). The impact of metal-induced stress on bacteria is revealed through measurable changes in cellular responses, notably expressed in alterations in gene transcription. These genetic changes are crucial for understanding metal resistance, defense mechanisms, and metal homeostasis in bacteria. Gaining insights into these genetic pathways is essential for effectively implementing efficient metal bioremediation and/ or biorecovery strategies (Choudhary and Sar, 2016; Wehrmann et al., 2019; Joudeh et al., 2021).

Despite extensive research on bacterial responses to metals, gaps remain in understanding the mechanisms of toxicity and resistance, especially for Y and other REEs. Specific information on the metabolic responses to these elements is scarce, highlighting the need for further research. Understanding how bacteria respond to Y at the gene transcriptional level can help identify key pathways involved in metal resistance. Bacteria employ various strategies to tolerate high metal concentrations., including efflux systems to remove the metal from cells, intracellular and/ or extracellular sequestration, and enzymatic detoxification to reduce toxicity (Hu et al., 2005; Maynaud et al., 2013; Wehrmann et al., 2019). For instance, metals like chromium and tellurium can be reduced less toxic metal forms, i.e., from Cr(IV) to Cr(III) and Te(IV)) to Te(0), respectively (Cervantes et al., 2001; Farias et al., 2021). Prokaryotes also have developed several complex molecular mechanisms to manage metal homeostasis, as reported in various studies concerning manganese (Mn) and zinc (Zn), such as the Zn uptake regulator Zur and the efflux pump *MntE* for maintaining Zn and Mn homeostasis, respectively (Feng et al., 2008; Turner et al., 2015; Xu et al., 2017; Grunenwald et al., 2019). However, similar mechanisms for Y have not been documented.

Transcriptomic data provide valuable insights into how changes in gene expression contribute to complex biological processes, including resistance mechanisms and adaptation to varying conditions (Choudhary and Sar, 2016; Huang et al., 2019; Wu et al., 2022). The mechanisms regulating Y homeostasis in *Mesorhizobium qingshengii* cells have not yet been explored, although similar studies exist for *Mesorhizobium metallidurans* in response to cadmium (Cd) and Zn (Maynaud et al., 2013).

In this study, the main objective was to assess the gene expression profile of the strain *M. qingshengii* J19 to identify the genes involved in the metabolic response that confers the strain’s high resistance to Y, and its ability to efficiently remove elevated concentrations of Y from the medium. Comparative genomic and transcriptomic analyses were employed to achieve this goal. This strain has previously demonstrated these remarkable abilities (Coimbra et al., 2024). Genome-wide transcriptional profiling was used to identify specific genes within the draft genome of strain *M. quingshengii* J19, focusing on those with significant differential expression after a 3 h exposure to 0.2 mM of Y. This approach provides insights into the strain’s molecular responses to Y, highlighting key pathways and mechanisms involved in its adaptive response to metal stress.

## 2 Materials and methods

### 2.1 Bacterial strain and growth condition

The strain *M. qingshengii* J19 was grown aerobically at 25°C on Reasoner’s 2A (R2Ab) liquid media (Himedia), containing per liter: 0.5 g of yeast extract, 0.5 g of proteose peptone, 0.5 g of casein, 0.5 g of glucose, 0.5 g of soluble starch, 0.3 g of K_2_HPO_4_, 0.024 g of MgSO_4_, and 0.3 g of sodium pyruvate. Analytical-grade yttrium salt (YCl_3_⋅6H_2_O) (Sigma-Aldrich) and iron(III) chloride anhydrous (FeCl_3_) (VWR) were prepared as a 0.5 M and 0.1M stock solution, respectively, and sterilized by filtration.

### 2.2 Genome sequencing and annotation

The bacterial strain J19 was grown in R2Ab liquid media, streaked from a single colony. Cells were collected, and the total DNA was extracted using E.Z.N.A.^®^ Bacterial DNA Kit (Omega Bio-Tek) according to manufacturer instructions.

Libraries of total genomic DNA were prepared using Nextera XT Preparation Kit (Illumina, San Diego, CA, United States) following the manufacturer’s instructions. Libraries were purified using HighPrep PCR Clean-up beads (MagBio Genomics, Inc.). Fragment analyzer 5200 (Agilent NGS Fragment 1-6000 pb methods) was used to analyze the fragment size distribution and molarity of each library. Nine-picomolar concentration libraries were sequenced on an Illumina MiSeq System based at the Section of Microbiology in the Department of Biology of Copenhagen University with 2 × 300bp chemistry (MiSeq Reagent Kit v3). Pairing, trimming, and assembly based on Bruijn graphs were performed using CLC Genomics Workbench v9.5.4 (Qiagen) using default parameters.

Genome annotation was performed upon submission to the GenBank databank using the NCBI Prokaryotic Genome Annotation Pipeline (PGAP) for the determination of coding DNA sequences (CDS) (Tatusova et al., 2016).

### 2.3 Structural and functional analysis of bacterial genome

The sequenced genome of strain J19 was functionally annotated in terms of a cluster of orthologous groups (COGs) using online eggnog-mapper v2 (Cantalapiedra et al., 2021).

### 2.4 Transcriptomes, RNA purification, sequencing and RNAseq analysis

#### 2.4.1 Yttrium stress

For the RNAseq analysis of strain J19, four biological replicates were pre-grown in R2Ab liquid media up to the mid-exponential phase, up to an optical density at 600 nm (OD_600nm_) between 0.3 and 0.4, in 250 ml Erlenmeyer flasks at 25°C and 140 rpm. When they reached the mid-exponential phase, the pre-cultures were treated with or without 0.2 mM of Y (treated cells and control, respectively) and incubated for 3 h, which corresponds to half a generation time, at 25°C at 140 rpm. After 3 h incubation, 4 ml of each bacterial culture cell was harvested by centrifugation at 4°C, 5000 *g* for 5 min. The supernatants were discarded, and the bacterial pellets were stored on ice until use. As described in our previous study, strain J19 can resist concentrations of Y up to 4 mM (Coimbra et al., 2024). The levels of Y in the abiotic controls with a Y concentration of 0.2 mM were previously studied and remained stable over time, indicating that the growth medium did not contribute to metal precipitation. In contrast, higher concentrations of Y showed some visible precipitates, but the Y levels did not present a statistically significant difference. Therefore, since 0.2 mM Y was one of the highest concentrations that did not show any precipitation in the culture medium and was the concentration that presented the highest immobilization of Y, it was selected for the RNAseq studies.

#### 2.4.2 RNA isolation

Total RNAs were purified from each replicate and each treatment using an enzymatic pretreatment with lysozyme (LYS), followed by the combined TRIzol^®^ (Invitrogen) and RNeasy^®^ mini kit (Qiagen) method, adjusted from the one described by Yu and colleagues (Yu et al., 2013). Each previous bacterial pellet was resuspended with 250 μl LYS (10 mg/ml prepared with PBS 1x) and incubated for 15 min at 37°C. For lysis to occur, 1 ml of TRIzol was added to the previous suspension and carefully resuspended with a micropipette. Subsequently, 200 μl of chloroform was added and the content of the tube was mixed well, incubated 10 min at room temperature before centrifugation at 4°C for 10 min at 12,000 g. The upper aqueous phase was collected and 250 μl of ethanol 70 % was added and mixed well. Each sample was then processed with RNeasy^®^ according to the manufacturer’s instructions. The total RNA was then treated with DNase I (Invitrogen) for 2 h at 37°C to remove residual DNA.

#### 2.4.3 Determination of RNA quantity and integrity

The RNA concentration was determined using Nanodrop 2000 (Thermo Scientific), and its integrity was checked by 1.2 % denaturing formaldehyde agarose (FA) gel electrophoresis. For the FA agarose gel preparation, 1.2 g of agarose was weighed and dissolved in 10 ml of 10x FA gel buffer (final concentration of 200 mM MOPS, 50 mM sodium acetate, and 10 mM EDTA, pH 7.0 and adjusted to 1 l of DEPC-treated water) and 90 ml of DEPC-treated water. The mixture was cooled and 1.8 ml of 37 % formaldehyde (Panreac) and 15 μl of 10 mg/ml ethidium bromide stock solution were added. A concentration of 200 ng RNA was prepared in 5x loading buffer (4 ml 10x FA gel buffer, 80 μl 0.5 M EDTA pH 8.0, 720 μl 37 % formaldehyde, 3.084 ml formamide, 2 ml 100 % glycerol, 16 μl bromophenol blue solution and adjusted to 10 ml with DEPC-treated water), in a proportion of 1 volume of 5x LB per 4 volume of sample, and loaded to the FA gel after incubation for 5 min at 65°C. Electrophoresis was performed at a constant current of 60 V for 1 h in a running buffer that consisted of 1x FA gel running buffer with 100 ml 10x FA gel buffer, 20 ml 37 % formaldehyde, and 880 ml DEPC-treated water. RNA integrity number (RIN) values were determined using a combination of agarose gel electrophoresis and rRNA ratio (23S:16S) calculation. After running the samples and staining the FA agarose gel, the gel was visualized using an imaging system. High-quality RNA is indicated by distinct ribosomal RNA bands (e.g., 23S and 16S rRNA for prokaryotes) with a clear separation between them. For a more quantitative assessment, the rRNA ratio was calculated by quantifying the intensity of the 23S and 16S rRNA bands using Quantity One^®^ analysis software. The ratio was obtained by dividing the intensity of the 23S band by that of the 16S band. A higher ratio indicates relatively intact RNA, while a lower ratio suggests degradation. For prokaryotic RNA, the rRNA ratio should be 2:1 for samples with intact RNA, which is indicative of good RNA quality. Finally, the RIN values were confirmed by Macrogen, Inc., (Seoul, South Korea) using a bioanalyzer to ensure accuracy.

#### 2.4.4 Library construction, RNA-seq and data pre-processing

A total RNA of 1 μg in every sample was used for library construction and only samples with a RIN value above 7 were accepted for sequencing. Macrogen, Inc., (Seoul, South Korea) carried out the sample sequencing, pre-processing, and analysis. For library preparation, the protocol for rRNA depletion was performed using the NEBNext rRNA Depletion Kit (Bacteria) (New England Biolabs). Following rRNA removal, the remaining RNA was purified, chemically fragmented, and randomly primed for reverse transcription (cDNA synthesis). Sequencing libraries were prepared with the TruSeq Stranded Total RNA Library Prep Gold Kit for Illumina (Reference Guide: 1000000040499 v00). The final products were subjected to sequencing on an Illumina NovaSeq™ 6000 platform with a paired-end 150 bp sequencing strategy (Illumina). From the raw sequence data obtained, the high–quality reads were filtered by eliminating low–quality reads with Q < 30 from the raw reads using FastQC (v0.11.9). The Trimmomatic program (v0.38) removes adapter sequences and bases with base quality lower than three from the ends (Bolger et al., 2014). Reads with lengths shorter than 36bp are dropped to produce trimmed data. The trimmed sequences obtained from RNA sequencing were mapped against the available reference annotated strain J19 draft genome using the short sequence alignment software Bowtie (v1.1.2) and, with the HTSeq program (v0.10.0), the read count per gene was extracted from the known gene annotations, which was used as the original raw data for further analysis.

#### 2.4.5 Differential gene expression analysis

Differential expression analysis requires that gene expression values should be compared among samples, performing the treated vs. control comparison pair. Therefore, during data pre-processing, the read count data was normalized with the relative log expression (RLE) method in the DESeq2 R library, where low-quality transcripts are filtered. Then, to proceed with a statistical test, RLE normalized count was adopted for the negative binomial Wald Test (nbinomWaldTest) in DESeq2. The significant results were selected on conditions of the absolute value of fold change (FC) above or equal to 1.5 (| FC| ≥ 1.5) and nbinomWaldTest raw *p*-value < 0.05.

### 2.5 Real-Time PCR analysis on selected gene

A further Real-Time PCR (RT-PCR) assay was conducted to validate the expression of the highest up-regulated gene obtained from RNA-seq results. Total RNA extractions from the middle of the exponential phase of strain J19 growth of control (no Y) and treated (supplemented with 0.2 mM of Y) were carried out using the GeneJet RNA Purification kit (Thermo Scientific), according to the manufacturer’s protocol and stored at −80°C until further use. The quality and concentration of total RNA isolated were measured using a Nanodrop 2000 spectrophotometer. The presence of any DNA contamination in the RNA samples was removed using DNase I, RNase-free (Thermo Scientific) for 2 h at 37°C, followed by heat inactivation with 50 mM EDTA at 65°C for 15 min. After DNase I treatment, RNA quality was checked by agarose gel electrophoresis and the absence of DNA contamination was confirmed by PCR. First-strand complementary DNA (cDNA) was synthesized from 200 ng of total RNA using SuperScript™ IV First-Strand cDNA synthesis Reaction (Invitrogen), according to the recommended protocol. cDNAs were prepared from three independent cultures and were further used for RT-PCR and each single-stranded DNA was quantified spectrophotometry using a Nanodrop 2000.

Transcriptional expression of the TonB-dependent receptor (CDS ID: J19_26075) was analyzed by RT-PCR. For each treatment, three different cDNA samples were considered, and each sample included three technical triplicates. Specific primers for the TonB-dependent receptor and the reference gene 16S rRNA, designed on the nucleotide sequences previously obtained with genome sequencing, were used, and are listed in Table 1. RT-PCR was performed on 10 μL final volume of 2x SYBR Green Master Mix (Bimake™) containing 700ng of cDNA using the Bio-Rad CFX96™ Real-Time PCR System. RT-PCR was carried out according to the following cycling program: 95°C for 5 min (hot start DNA polymerase activation), 40 cycles at 95°C for 30 s and 57°C for 30 s (denaturation and annealing), and the dissociation curve step at 95°C for 15 s, 60°C for 1 min and 95°C for 15 s. No genomic contamination was detected by the dissociation curve. Relative quantification values were obtained using the Pfaffl mathematical model (2^–ΔΔCt^ calculation) (Pfaffl, 2001), where values obtained for treated samples were compared with those obtained for untreated samples (control). The amounts of expression levels were normalized to the reference gene.

**TABLE 1:** Primers used in this study with description of annealing temperature used in RT-PCR, amplicon length and sequence.

Amplicon	Primer	Sequence (5′ – 3′)	Amplicon length (bp)	Annealing temperature (°C)
16S rRNA	357-F 534-R	TACGGGAGGCAGCAG ATTACCGCGGCTGCTGG	178	57
TonB-dependent receptor (CDS ID: J19_26075)	tonB-F tonB-R	TGACCAGCCTCGTCCCGCTCA AGTCTCGACGTCATCATTCAC	194	57

### 2.6 ROS quantification and iron supplementation’s effect

The ability of strain J19 to grow in a Y-supplemented R2Ab medium was evaluated to determine the toxic concentration of the metal for the cells in its growth kinetic. The strain was grown in the R2Ab medium in the presence or absence of a concentration of Y (0.4 mM), as described above. The inoculated medium without Y was used as a control. At appropriate incubation times, bacterial growth was determined by OD_600nm_ measurements.

The oxidative stress was determined by measuring the formation of ROS by using 2,7-dichlorofluorescein-diacetate (H_2_DCFDA) (Invitrogen) assay (Oparka et al., 2016). J19 cells were grown in R2Ab supplemented with and without Y at a concentration of 0.4 mM. After 14 h of growth, corresponding to the middle exponential phase, cultures were washed twice with phosphate buffer saline (PBS) solution (containing per liter: 8 g NaCl, 0.2 g KCl, 1.44 g Na_2_HPO_4_, 0.24 g KH_2_PO_4_, pH 7.4), adjusted to an OD_600nm_ of 0.3 and then incubated in 25 μM of H_2_DCFDA for 1 h at 25°C. Cells were retrieved and once again washed twice with PBS solution, and the pellets were resuspended in 1 mL PBS. To determine the level of ROS, the fluorescence (λem = 527 nm and λex = 495 nm) and OD_600nm_ were read hourly for 12 h. Additionally, to evaluate the impact of iron on bacterial growth, as well as on ROS production, in the presence of Y, J19 cells were grown in R2Ab supplemented with 0.4 mM of Y and 100 μM of Fe. The results were expressed as relative fluorescence units (RFU), calculated as the ratio of fluorescence to the optical density. Assays were conducted at least in duplicate.

### 2.7 Statistical analysis

Each result is presented as the mean value of two independent experiments (the number of independent experiments is indicated in the caption of each figure) ± the standard derivation. Statistical analysis was performed using GraphPad Prism 9 for Windows using Ordinary one-way ANOVA followed by Tukey’s multiple comparisons tests. All differences were considered statistically significant for *p*-value < 0.05.

### 2.8 Data availability

The datasets presented in this study can be found in online repositories. The strain is available at the University of Coimbra Bacteria Culture Collection (UCCCB) under the identification number UCCCB 155. The draft genome of strain J19 was deposited in the National Center for Biotechnology Information (NCBI) public database (Bethesda, MD, USA) under accession number JAPFQA000000000. The RNAseq data are available from NCBI GEO datasets under the accession number PRJNA1048157.

## 3 Results and discussion

### 3.1 Genome sequencing, assembly and automated annotation

**FIGURE 1:**
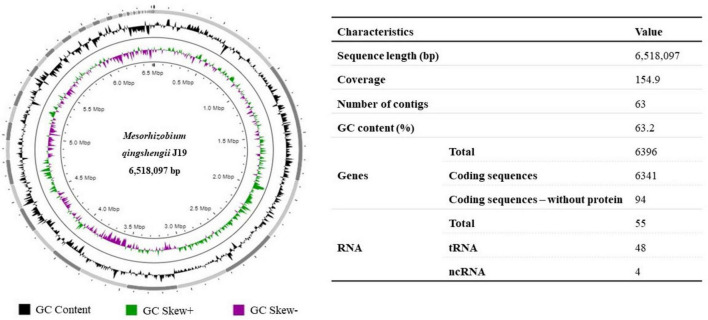
Graphical circular representation of the draft genome of *M. qingshengii* J19 using Proksee (Grant et al., 2023). Circles from exterior to interior represents: GC content (black), GC Skew+ which indicates G > C (green) and GC Skew–which indicates G < C (purple). The table on the right shows the predicted basic genomic features of *M. qingshengii* J19.

The draft genome of *M. qingshengii* J19 was obtained by Illumina sequencing to acquire the genetic information of this Y-resistant strain, which was then used as a reference for mapping the transcriptome sequence reads. The general characteristics of the strain’s J19 genome are summarized in Figure 1. The draft genome sequence comprised 6,518,097 bp and is assembled into 63 contigs, with an average GC content of 63.2 %. A total of 6,341 CDS and 55 RNAs, including 48 tRNA and 4 ncRNAs, were predicted.

### 3.2 Analysis of orthologous genes

The genes of *M. qingshengii* obtained from Illumina sequencing were analyzed using the clusters of orthologous groups (COG) database to classify them and predict their functions. The COG annotation revealed that 74.1 % (4,631) of the coding sequences were matched to putative proteins with known functions and assigned to 21 COG categories (Table 2). The most representative functional category was metabolism processes, with 58.4 % of the genes. Within this COG category, class E (amino acid metabolism and transport) was the most predominant functional category, representing 15.9 % of the genes, followed by classes G (carbohydrate metabolism and transport), P (inorganic ion transport and metabolism), and C (energy production and conversion), which accounted for 10.4 %, 8.1 % and 7.7 % of the genes, respectively. The remaining categories, cellular processes and signaling, and information storage and processing processes, represented about 22.0 % and 19.9 % of the COG categories, respectively.

**TABLE 2:** COG functional assignment of CDS detected in the draft genome of strain J19.

COG class ID	Process	Description	CDS number*
E	Metabolism	Amino Acid metabolism and transport	735
G		Carbohydrate metabolism and transport	482
P		Inorganic ion transport and metabolism	377
C		Energy production and conversion	357
I		Lipid metabolism	267
Q		Secondary Structure	212
H		Coenzyme metabolism	177
F		Nucleotide metabolism and transport	97
K	Information storage and processing	Transcription	520
J		Translation	207
L		Replication and repair	190
B		Chromatin Structure and dynamics	5
M	Cellular processes and signaling	Cell wall/membrane/envelop biogenesis	312
T		Signal Transduction	237
O		Post-translational modification, protein turnover, chaperone functions	208
U		Intracellular trafficking and secretion	85
V		Defense mechanisms	73
D		Cell cycle control and mitosis	51
N		Cell motility	46
Z		Cytoskeleton	4
W		Extracellular structures	1

* 4,631 CDS out of 6,247 (74.1 %) were assigned to at least one COG class ID.

These results align with previous genome annotations of various *Mesorhizobium* species. Compared to the strain J19, strains *M. australicum* (Reeve et al., 2013a) and *M. opportunistum* (Reeve et al., 2013b) had a lower percentage of coding genes with assigned functions, around 56 %. This suggests that strain J19 has a higher proportion of CDS classified into COG functional categories, indicating a more thoroughly annotated genome with better-characterized functional capabilities. Nevertheless, similar to strain J19, the most prevalent functional category in these strains was metabolism, with around 44 % of genes in this category, with class E being the most outstanding. Additionally, classes M and K were the primary representatives in the cellular processes and signaling, and information storage and processing categories, respectively, as observed in strain J19.

### 3.3 Differential gene expression under Y stress

To understand the mechanisms by which *M. qingshengii* J19 responds to Y toxicity, we compared the transcriptional profile of the strain under Y treatment to those of untreated controls using RNAseq analysis. The experiment included two conditions: Y treatment (referred to as the treated condition) and no Y treatment (referred to as the control condition), with four biological replicates for each. The general characteristics of the RNAseq data are summarized in Table 3, demonstrating consistency between samples and indicating that observed differences in gene expression are attributable to experimental conditions rather than technical variability or sample inconsistency. After quality control and trimming, an average of 72,745,193.5 reads for control cells and 81,678,806 reads for treated cells were obtained. RNAseq reads were aligned against the 63 contigs of the reference draft genome, with approximately 52 % of the reads mapping to specific contigs.

**TABLE 3:** Summary of sequencing data quality from transcriptome libraries of *M. qingshengii* J19 with or without Y exposure.

		Total read bases^a^	Total reads	Total clean reads^b^	GC (%)	Q20 (%)^c^	Mapped reads (%)^d^
Control	J19_1	10,168,011,424	67,337,824	65,538,216	61.67	98.83	43.26
	J19_2	12,958,761,110	85,819,610	83,187,496	62.06	98.89	60.89
	J19_3	10,341,387,510	68,486,010	66,021,780	62.24	98.89	53.07
	J19_4	11,800,536,750	78,149,250	76,233,282	61.35	98.86	52.94
Treated	J19 Y_1	11,805,307,746	78,180,846	75,987,694	62.01	98.87	49.01
	J19 Y_2	12,909,510,044	85,493,444	83,813,776	61.17	98.84	51.27
	J19 Y_3	12,989,176,738	86,021,038	84,287,048	62.28	98.90	56.27
	J19 Y_4	12,715,585,274	84,209,174	82,626,706	62.07	98.88	51.64

^a^ Total number of bases sequenced.

^b^ Total number of reads after trimming.

^c^ Ratio of bases that have a phred quality score greater than or equal to 20.

^d^ Number of reads mapped to reference.

Out of a total of 6,389 genes, 6,343 genes were included in the statistical analysis after excluding 46 genes with at least one zero count. Distributional characteristics for raw and normalized expression counts are provided in the Supplementary Figure 1. The raw data showed a median expression bias and density distribution (Supplementary Figures 1A, B). Following normalizing using the RLE method in DESeq2, both the boxplot and the density distribution achieved homogeneity (Supplementary Figures 1C, D), with no outlier samples and comparable data distribution between samples.

**FIGURE 2:**
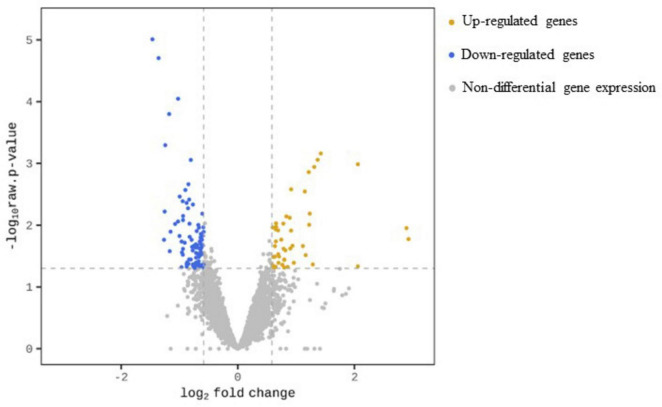
Volcano plot for transcription profile of *M. qingshengii* J19 in the presence of Y, representing the non-DGEs (grey circles) and DGEs of up-regulated (yellow circles) and down-regulated genes (blue circles). Genes with a | FC| ≥ 1.5 and an adjusted *p*-value < 0.05 were defined as differential gene expression.

Following Y treatment, a total of 127 genes, representing 2% of the genes mapped to our reference draft genome, were significantly differentially expressed (| FC| ≥ 1.5, *p*-value < 0.05). Among these, 46 genes were up-regulated and 81 genes were down-regulated, accounting for 36.2 % and 63.8 % of the total number of differential gene expressions (DGEs), respectively. The results are summarized in Figure 2.

### 3.4 Functional composition of J19 transcriptomes and change upon Y exposure

**FIGURE 3:**
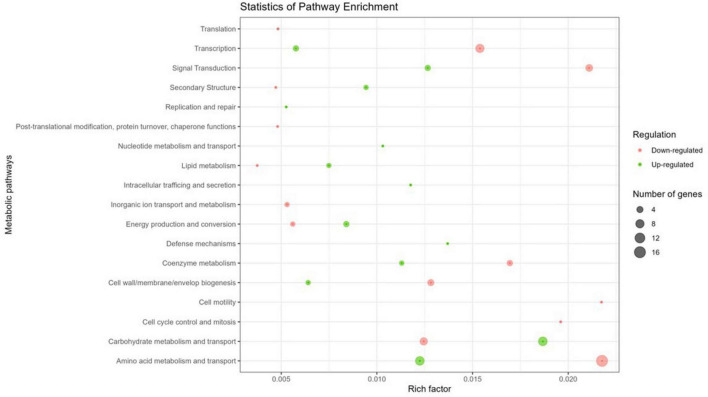
Circos plot illustrating the top 18 COG functions of DGEs in the transcriptome of *M. qingshengii* J19 under Y stress (created with R package). The first circle: 18 COG pathways indicated by the class ID. Different colors represent different COG categories. The second circle: a bar chart of the proportion of up- and down-regulated genes in percentage. Green represents the proportion of up-regulated genes, and red is the proportion of down-regulated genes. The third circle: number of genes per COG pathway.

To assess the impact of Y stress on metabolic pathways, the 127 DGEs were grouped based on their COG pathways to identify functional differences in gene expression. Of the identified genes, 72.4 % of coding sequences were assigned known functions and classified into 18 COG categories (Figure 3). Specifically, 64.1 % of the genes were associated with metabolic processes, 20.7 % with cellular processes and signaling, and the remaining 15.2 % with information storage and processing. Within these categories, the top 2 metabolic pathways were classes E (amino acid metabolism and transport; 42.4 %) and G (carbohydrate metabolism and transport; 25.4 %) for the metabolism category, classes K (transcription; 78.6 %) and J (translation; 14.3 %) for the information storage and processing category, and classes T (signal transduction; 42.1 %) and M (cell wall/membrane/envelope biogenesis; 31.6 %) for the cellular processes and signaling category. Several studies have demonstrated that bacteria exposed to metals are affected in similar pathways, in the same preferential order as observed with Y. For example, *E. coli* exposed to Pd, and *M. metallidurans* STM 2683T and *Mesorhizobium* sp. exposed to Cd and Zn show comparable pathway disruptions (Maynaud et al., 2013; Joudeh et al., 2021).

**FIGURE 4:**
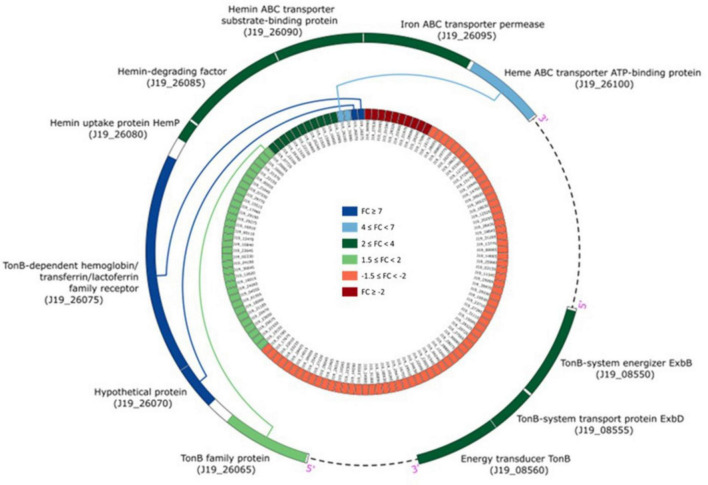
Yttrium impact on metabolic pathways representation in *M. qingshengii* J19 for the 92 DGEs with an assigned metabolic function (out of a total of 127 DGEs). The size of the dot indicates the number of DGEs enriched in the metabolic pathway, and the color indicates the up- and down-regulated genes (blue and red, respectively). Rich factor was defined as the ratio of the number of the DGEs enriched in the pathway to the number of all genes annotated to this pathway.

The genes that were detected and identified as being part of a functional pathway were analyzed to determine whether the metabolic pathways in which they are involved were activated or deactivated. The significance of each pathway’s activation or deactivation was assessed based on the proportion of genes detected relative to the pathway’s size (total number of genes in the pathway present in the reference transcriptome) (Figure 4). In this analysis, a cut-off value, known as the rich factor, was set at a maximum of 0.025. In cells exposed to 0.2 mM of Y, the most significantly enriched COG pathway associated with positive DGE was carbohydrate metabolism and transport, with 9 genes and a rich factor 1.5-fold higher than the negative DGEs. On the other hand, the most significantly enriched pathways associated with negative DGEs included amino acid metabolism and transport (16 genes), transcription (8 genes), signal transduction (5 genes), coenzyme metabolism (3 genes), cell motility (1 gene) and cell cycle control and mitosis (1 gene). Notably, pathways related to cell mobility and cell cycle control and mitosis were exclusively enriched in negative DGEs. Furthermore, other pathways had rich factors up to 2.7, 1.8, 1.7, and 1.5 times higher for transcription, amino acid metabolism and transport, signal transduction, and coenzyme metabolism, respectively, compared to positive DGEs.

The CDS presenting *p*-values below 0.05 and an absolute FC of 1.5 or higher in the “Treated” vs “Control” experiment are listed in the Supplementary Tables 1, 2, which list up- and down-regulated genes, respectively. Among the differentially over-expressed genes (FC > 4) identified in our study, three notable ones related to iron homeostasis are CDS J19_26075, CDS J19_26070, and CDS J19_26100. These genes encode for the TonB-dependent hemoglobin/transferrin/lactoferrin family receptor, a hypothetical protein, and the heme ABC transporter ATP-binding protein, respectively. The last two CDS genes were up-regulated by 4.2- and 7.4-fold, while the first was remarkably up-regulated by 7.6-fold. These findings highlight the significant up-regulation of genes associated with iron homeostasis in response to Y stress.

**FIGURE 5:**
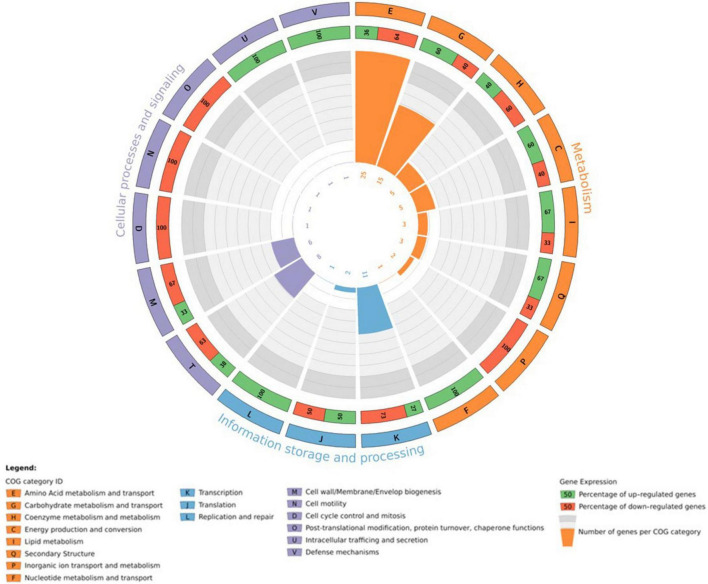
Circos plot illustrating the integration of transcriptomic information in the genomic arrangement of *M. qingshengii* J19 for the genes involved in iron homeostasis (FC > 1.5) (created with R package).

Iron is an essential micronutrient for most microorganisms, acting as a cofactor in several enzymes and as a catalyst in electron transport processes (Chiancone et al., 2004; Kim et al., 2009; Bradley et al., 2020). Using the STRING database (Szklarczyk et al., 2023), it was possible to predict that CDS J19_26070 is associated with a TonB-related protein. A detailed analysis of genes co-located in the genome of strain J19 highlighted several genes involved in iron homeostasis that are significantly over-represented with a *p*-value > 0.05, as shown in Figure 5. Genes adjacent to the overexpressed gene (CDS J19_26070) are also involved in iron homeostasis, showing up-regulation ranging from 2.8 to 3.7 (*p*-value > 0.05). Additionally, another group of iron homeostasis genes, located in a different region of the draft genome, exhibit up-regulation between 2.7 and 3.1 (*p*-value > 0.05). These genes code for the energy transducer TonB (CDS J19_08560), the TonB-system transporter protein ExbD (CDS J19_08555), and the TonB-system energizer ExbB (CDS J19_08550). In Gram-negative bacteria as strain J19, iron acquisition requires outer membrane-localized proteins that bind iron at the cell surface and facilitate its uptake. These TonB-dependent receptors require energy from the proton motive force and the periplasmic proteins complex ExbD-ExbB-TonB to transport this energy to the outer membrane (Moeck and Coulton, 1998; Andrews et al., 2003; Caldeira et al., 2021).

The up-regulation of the highest overexpressed gene, the TonB-dependent receptor, in strain J19, along with the proteins ExbD (CDS J19_08555), ExbB (CDS J19_08550), and the energy transducer TonB (CDS J19_08560), suggests a potential resistance mechanism in response to Y-induced toxicity. These genes are important for iron transport and may play a significant role in Y resistance and cellular protection against elemental toxicities. Studies on different metals have demonstrated diverse effects on genes encoding homologous of the TonB-ABC transport system. For example, exposure to excess lanthanides has been shown to downregulate this system, affecting genes within the lut cluster (Wegner et al., 2021). Conversely, nickel exposure leads to the upregulation of TonB-dependent receptor-like proteins (Volpicella et al., 2017). These findings highlight that the transcriptional response of these genes is dynamic and influenced by the specific metal involved and the bacterial strain’s characteristics. Further investigation into the role of these genes in the Y detoxification strategy by strain J19 was conducted to validate their involvement.

In addition to genes related to iron homeostasis, another over-expressed gene involved in an alternative detoxifying mechanism was identified (CDS J19_31980). This gene encodes arsenate reductase ArsC, which was greatly up-regulated by 4.2-fold. ArsC protein functions by converting intracellular As(V) into As(III), which is then exported from the cells through an energy-dependent efflux process (Dhankher et al., 2003). These findings suggest that Y exposure triggers a cellular stress response, activating non-Y-specific response systems, such as the arsenic detoxifying system. Additionally, Dhankher and colleagues (Dhankher et al., 2003) demonstrated that ArsC can confer cadmium (Cd) tolerance in *E. coli*. Furthermore, Maertens and colleagues (Maertens et al., 2020) have shown that exposure of *Cupriavidus metallidurans* cells to copper (Cu) has led to the activation of multiple genes from the *ars* cluster.

On the other hand, no significant differences were observed in the expression of down-regulated genes, which had FC values ranging between −1.5 and −2.8 (Supplementary Table 2). Exposure to heavy metals, whether essential and non-essential, can cause toxicity when present at high concentrations, leading to oxidative stress and nucleic acid damage through the production of ROS.

### 3.5 RT-PCR validation

The gene encoding the TonB-dependent receptor (CDS J19_26075) was identified as the most highly overexpressed under Y stress. To validate this finding, real-time PCR (RT-PCR) was performed. This step was essential not only to confirm the accuracy of the RNAseq data but also to verify the significance of this protein in the mechanism of Y resistance. The 16S rRNA gene was selected as the reference gene, as its expression remained unaltered by metal treatment. The relative expression levels of the target gene, as measured by RT-PCR, were compared with those obtained from the RNAseq approach (Supplementary Table 3). The RT-PCR results revealed a significant increase in the expression of the gene TonB-dependent receptor under Y stress, with a ratio of 3.9. These results corroborate the RNAseq data and further support the role of the TonB-dependent receptor in the bacterial response to Y stress.

### 3.6 Effect of iron supplementation on Y stress

**FIGURE 6:**
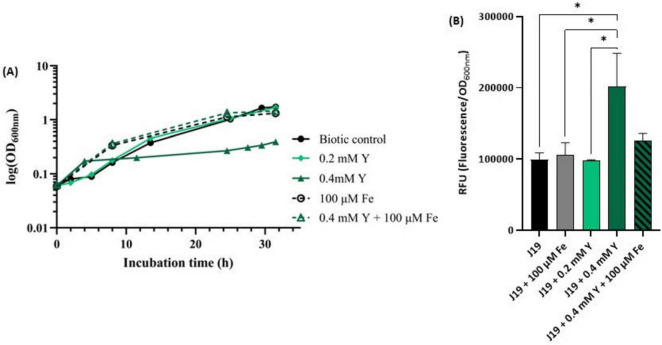
Growth curve of strain *M. qingshengii* J19 measured over time in R2Ab liquid medium supplemented with 0.2 and 0.4 mM Y and/ or 100 μM Fe **(A)**, and ROS production **(B)**. A biotic control, i.e., without metal, is present for both assays. Means ± standard deviations - error bars - were calculated from two independent experiments, including the growth curve assay. *Indicates a significant difference with *p*-value < 0.05.

The results strongly indicate that iron plays a significant role in the Y-bacterial response mechanism. To further investigate this, assays were conducted to assess the impact of free Fe^3+^ on the growth of strain J19 exposed to high concentrations of Y, known to be toxic to cells (Figure 6A). The highest concentration of Y used, 0.4 mM, drastically impacted the growth of strain J19, causing a 4-fold decrease in maximum OD_600nm_ compared to the control (without Y). Supplementing the growth medium with 100 μM Fe alone improved bacterial growth compared to the control. However, when Fe was combined with the toxic concentration of Y, it reversed the growth inhibition caused by Y. Under this co-supplementation, bacterial growth increased 5-fold compared to growth in the presence of Y alone, and also showed enhancement compared to both controls, without any metal and with Fe alone. Additionally, no difference was observed in bacterial growth between the cultures when supplemented with 100 μM Fe alone and combined with 0.4 mM Y.

These results highlight the protective role of iron in mitigating the toxic effects of Y on strain J19. As far as we know, there are no specific studies reporting Y resistance or detoxification through iron supplementation in bacterial species. However, similar interactions with other metals have been explored. For example, an indium(In)-sensitive mutant of strain *Rhodanobacter* sp. B2A1Ga4, which is sensitive to both In and gallium (Ga), showed that iron supplementation reversed the growth inhibition caused by these metals (Caldeira et al., 2021). The same sensitivity was observed with *Pseudomonas fluorescens* in the presence of In (Anderson and Appanna, 1993). Moreover, Steunou and colleagues (Steunou et al., 2020) demonstrated that the resistance of several bacterial strains to metals like Cu and Cd relied on the expression of Fe transporters.

The toxic concentration of Y (i.e., 0.4 mM) was also evaluated for its effect on ROS production in strain J19 (Figure 6B). The results revealed that strain J19 produced up to 2-fold more ROS when exposed to the highest concentration of Y compared to the control. The addition of iron notably reduced ROS production in cells exposed to Y, decreasing ROS levels by up to 1.6-fold, thereby approaching baseline values. These findings suggest that iron plays a crucial role in the detoxification of ROS induced by Y. This is consistent with observations by Caldeira and colleagues (Caldeira et al., 2021) in bacterial cells exposed to Ga and In, as well as studies in *Vibrio cholerae*, *E. coli* and *Rubrivivax gelatinosus*, where metal excess, such as Cu or Cd, induced Fe uptake and activated the ROS detoxification system (Steunou et al., 2020).

## 4 Conclusion

The results of this study greatly improve our understanding of how the highly resistant bacterium *M. qingshengii* J19 responds to Y exposure at both cellular and metabolic levels. Genome sequencing and transcriptomics analysis have revealed, for the first time, the specific metabolic changes in strain J19 in response to Y, providing new insights into its resistance mechanisms. The study identified multiple differentially overexpressed genes, particularly those involved in iron homeostasis, highlighting their crucial role in Y resistance. Moreover, strain J19 activated non-Y-specific mechanisms under Y exposure. At high concentrations, Y increases cellular ROS production, which limits the growth of strain J19. However, iron supplementation is essential for controlling ROS levels and protecting cells from Y toxicity.

## Data Availability

The datasets presented in this study can be found in online repositories. The names of the repository/repositories and accession number(s) can be found below: NCBI-JAPFQA000000000, PRJNA1048157.
