# Supplementary material for: Iron homeostasis as a cell detoxification mechanism in Mesorhizobium qingshengii J19 under yttrium exposure
Source: Front Microbiol. 2024 Oct 4;15:1467386. doi: 10.3389/fmicb.2024.1467386 (PMC11486727; doi:10.3389/fmicb.2024.1467386)
Supplement: Supplementary file 1 [file Data_Sheet_1.docx]

***Supplementary Material***

Iron homeostasis as a cell detoxification mechanism in *Mesorhizobium qingshengii* J19 under yttrium exposure

**Carina Coimbra, Rita Branco, Paula V. Morais^1*^**

*** Correspondence:** Paula V. Morais: pvmorais@ci.uc.pt

# Supplementary Tables

**Supplementary Table 1.** List of *M. qingshengii* J19 CDS differentially up-regulated upon Y exposure, grouped according to the COG functional group assigned.

| **COG category / pathway** | **CDS ID** | **Description** | **Treated / Control** | |
| --- | --- | --- | --- | --- |
|  |  |  | **FC** | ***p*-value** |
| Metabolism | | | | |
| Amino Acid metabolism and transport | **J19_26100** | **heme ABC transporter ATP-binding protein** | **4,2** | **0,046** |
|  | J19_22155 | hypothetical protein | 1,9 | 0,024 |
|  | J19_29770 | tetraacyldisaccharide 4’-kinase | 1,8 | 0,026 |
|  | J19_17460 | uroporphyrinogen-III C-methyltransferase | 1,7 | 0,049 |
|  | J19_29190 | cobalamin-binding protein | 1,7 | 0,037 |
|  | J19_16840 | low molecular weight phosphatase family protein | 1,6 | 0,032 |
|  | J19_24190 | nicotinate-nucleotide adenylyltransferase | 1,6 | 0,012 |
|  | J19_30045 | heavy metal translocating P-type ATPase | 1,6 | 0,011 |
|  | J19_01355 | SDR family oxidoreductase | 1,6 | 0,022 |
| Carbohydrate metabolism and transport | **J19_26070** | **hypothetical protein** | **7,4** | **0,011** |
|  | J19_15995 | peptidase M75, Imelysin | 2,7 | 0,001 |
|  | J19_16000 | DUF1513 domain-containing protein | 2,6 | 0,001 |
|  | J19_15990 | thiol oxidoreductase | 2,5 | 0,001 |
|  | J19_26340 | hypothetical protein | 2,2 | 0,003 |
|  | J19_29770 | tetraacyldisaccharide 4’-kinase | 1,8 | 0,026 |
|  | J19_15515 | formate dehydrogenase accessory sulfurtransferase FdhD | 1,7 | 0,036 |
|  | J19_00110 | FAD-binding protein | 1,7 | 0,044 |
|  | J19_24265 | NADPH-dependent oxidoreductase | 1,6 | 0,012 |
| Coenzyme metabolism | J19_16005 | tRNA (adenosine(37)-N6)-threonylcarbamoyltransferase complex dimerization subunit type 1 TsaB | 1,9 | 0,003 |
|  | J19_12470 | metalloregulator ArsR/SmtB family transcription factor | 1,7 | 0,017 |
| Energy production and conversion | J19_07725 | DEAD/DEAH box helicase | 1,9 | 0,041 |
|  | J19_12620 | ABC transporter ATP-binding protein | 1,6 | 0,009 |
|  | J19_18060 | DNA repair protein RecN | 1,6 | 0,048 |
| Lipid metabolism | J19_00555 | hypothetical protein | 1,9 | 0,008 |
|  | J19_04555 | ATP-binding cassette domain-containing protein | 1,6 | 0,047 |
| Nucleotide metabolism and transport | J19_02330 | DUF1284 domain-containing protein | 1,6 | 0,029 |
| Secondary structure | J19_00555 | hypothetical protein | 1,9 | 0,008 |
|  | J19_04555 | ATP-binding cassette domain-containing protein | 1,6 | 0,047 |
| Information storage and processing | | | | |
| Replication and repair | J19_23650 | citrate synthase | 1,5 | 0,032 |
| Translation | J19_20675 | LacI family transcriptional regulator | 1,5 | 0,011 |
| Transcription | J19_06995 | HlyD family efflux transporter periplasmic adaptor subunit | 2,3 | 0,010 |
|  | J19_13335 | aspartate/glutamate racemase family protein | 2,2 | 0,031 |
|  | J19_01570 | ROK family protein | 1,9 | 0,012 |
| Cellular processes and signaling | | | | |
| Cell wall/membrane/envelop biogenesis | J19_25240 | ABC transporter ATP-binding protein | 2,3 | 0,007 |
|  | J19_23645 | citrate synthase/methylcitrate synthase | 1,6 | 0,041 |
| Defense mechanisms | J19_21045 | cupin domain-containing protein | 1,8 | 0,047 |
| Intracellular trafficking and secretion | J19_07330 | dephospho-CoA kinase | 1,8 | 0,007 |
| Signal Transduction | **J19_26075** | **TonB-dependent hemoglobin/transferrin/lactoferrin family receptor** | **7,6** | **0,017** |
|  | **J19_31980** | **arsenate reductase ArsC** | **4,2** | **0,001** |
|  | J19_16010 | ribosomal protein S18-alanine N-acetyltransferase | 1,7 | 0,009 |

Gene IDs on bolt were the most over-expressed genes under Y stress with FC > 4.

**Supplementary Table 2.** List of *M. qingshengii* J19 CDS differentially down-regulated upon Y exposure, grouped according to the COG functional group assigned.

| **COG category / pathway** | **CDS ID** | **CDS description** | **Treated / Control** | |
| --- | --- | --- | --- | --- |
|  |  |  | **FC** | ***p*-value** |
| Metabolism | | | | |
| Amino Acid metabolism and transport | J19_17675 | GGDEF domain-containing protein | -1,5 | 0,01080 |
|  | J19_03235 | hypothetical protein | -1,5 | 0,01485 |
|  | J19_06645 | class I SAM-dependent methyltransferase | -1,5 | 0,02081 |
|  | J19_22305 | DUF3309 family protein | -1,6 | 0,04418 |
|  | J19_19290 | ABC transporter permease | -1,6 | 0,01385 |
|  | J19_04240 | GGDEF domain-containing protein | -1,6 | 0,02104 |
|  | J19_28885 | hypothetical protein | -1,6 | 0,01243 |
|  | J19_27395 | phosphoketolase family protein | -1,7 | 0,04563 |
|  | J19_30970 | DUF2147 domain-containing protein | -1,7 | 0,02244 |
|  | J19_18630 | anti-sigma factor | -1,8 | 0,04683 |
|  | J19_30020 | RcnB family protein | -1,9 | 0,00269 |
|  | J19_19775 | hypothetical protein | -2,0 | 0,04795 |
|  | J19_17090 | hypothetical protein | -2,0 | 0,00871 |
|  | J19_20245 | nitrate reductase subunit beta | -2,1 | 0,00954 |
|  | J19_01970 | aminomethyltransferase family protein | -2,2 | 0,02638 |
|  | J19_06960 | rubrerythrin family protein | -2,8 | 0,00001 |
| Carbohydrate metabolism and transport | J19_31380 | sulfite exporter TauE/SafE family protein | -1,6 | 0,02531 |
|  | J19_22005 | PilZ domain-containing protein | -1,6 | 0,02308 |
|  | J19_04240 | GGDEF domain-containing protein | -1,6 | 0,02104 |
|  | J19_29325 | zinc ABC transporter permease AztB | -1,6 | 0,02311 |
|  | J19_29330 | zinc ABC transporter ATP-binding protein AztA | -1,7 | 0,02565 |
|  | J19_14755 | cbb3-type cytochrome c oxidase subunit 3 | -1,9 | 0,01921 |
| Coenzyme metabolism | J19_14015 | ABC transporter substrate-binding protein | -1,5 | 0,04796 |
|  | J19_27395 | phosphoketolase family protein | -1,7 | 0,04563 |
|  | J19_15170 | OmpA family protein | -1,9 | 0,00826 |
| Energy production and conversion | J19_14015 | ABC transporter substrate-binding protein | -1,5 | 0,04796 |
|  | J19_08995 | hypothetical protein | -1,8 | 0,00384 |
| Inorganic ion transport and metabolism | J19_28885 | hypothetical protein | -1,6 | 0,01243 |
|  | J19_27030 | hypothetical protein | -2,6 | 0,00002 |
| Lipid metabolism | J19_03360 | hypothetical protein | -2,3 | 0,00016 |
| Secondary Structure | J19_03360 | hypothetical protein | -2,3 | 0,00016 |
| Information storage and processing | | | | |
| Translation | J19_03135 | SH3 domain-containing protein | -1,7 | 0,00088 |
| Transcription | J19_28425 | hypothetical protein | -1,5 | 0,04385 |
|  | J19_27220 | rhodanese-like domain-containing protein | -1,5 | 0,02302 |
|  | J19_10265 | DUF1153 domain-containing protein | -1,6 | 0,02760 |
|  | J19_29470 | transporter substrate-binding domain-containing protein | -1,6 | 0,04337 |
|  | J19_16755 | helix-turn-helix domain-containing protein | -1,6 | 0,03241 |
|  | J19_29910 | EAL domain-containing protein | -1,6 | 0,01094 |
|  | J19_31295 | polyphosphate kinase 2 | -1,8 | 0,04223 |
|  | J19_18625 | sigma-70 family RNA polymerase sigma factor | -1,9 | 0,02765 |
| Cellular processes and signaling | | | | |
| Cell cycle control and mitosis | J19_10285 | cell cycle two-component system response regulator CtrA | -1,6 | 0,01838 |
| Cell motility | J19_04630 | hypothetical protein | -1,6 | 0,03823 |
| Cell wall/membrane/envelop biogenesis | J19_28890 | murein L,D-transpeptidase | -1,6 | 0,03063 |
|  | J19_22005 | PilZ domain-containing protein | -1,6 | 0,02308 |
|  | J19_01580 | dicarboxylate/amino acid:cation symporter | -2,4 | 0,00601 |
|  | J19_01995 | NAD(P)/FAD-dependent oxidoreductase | -2,4 | 0,01722 |
| Post-translational modification, protein turnover, chaperone functions | J19_15175 | DUF1203 domain-containing protein | -2,0 | 0,00009 |
| Signal Transduction | J19_16755 | helix-turn-helix domain-containing protein | -1,6 | 0,03241 |
|  | J19_15245 | PRC-barrel domain-containing protein | -1,6 | 0,04388 |
|  | J19_23710 | RNA polymerase factor sigma-32 | -1,7 | 0,04288 |
|  | J19_11345 | hypothetical protein | -1,7 | 0,03589 |
|  | J19_30020 | RcnB family protein | -1,9 | 0,00269 |

**Supplementary Table 3.** Comparison of gene expression levels by RNAseq and RT-PCR of TonB-dependent receptor (CDS ID: J19_26075) from *M. qingshengii* J19. Expression level ratio of the target gene (treated with 0.2 mM Y compared to control). Values were calculated by the Pfaffl method using the 16S rRNA gene as the reference gene.

|  | **RNAseq** | **RT-PCR** |
| --- | --- | --- |
| ***M. qingshengii* strain J19 with 0.2 mM Y** (J19_26075) | 7.6 ± 0.02 | 3.9 ± 0.65 |

# Supplementary Figures

**
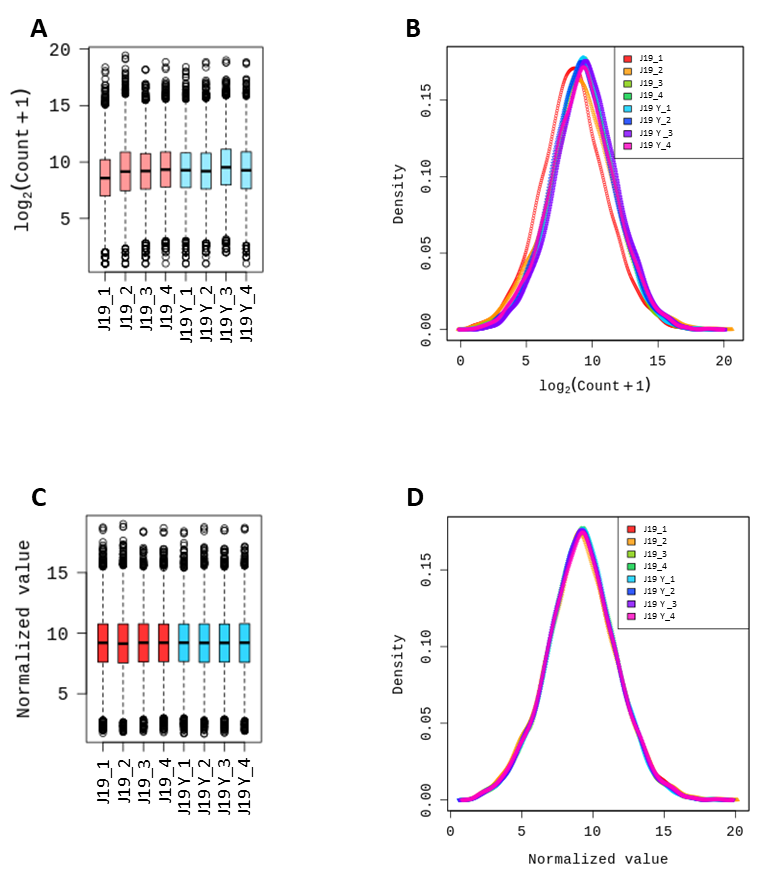
**

**Supplementary Figure 1.** Quality control of RNAseq transcriptomic data. Box plot and density distribution for un-normalized data (**A** and **B**, respectively), and normalized data with the RLE method using the DESeq2 (**C** and **D**, respectively).
